# Nuclear damage-induced DNA damage response coupled with IFI16-driven ECM remodeling underlies dilated cardiomyopathy

**DOI:** 10.7150/thno.112247

**Published:** 2025-04-28

**Authors:** Qingyong He, Xing Chang, Hui Zhang, Qianying Hao, Jianguo Zhi, Hongshuo Shi, Yingjie Tian, Hao Zhou, Ying Tan, Junmeng Zheng, Junxiong Qiu, Jun Tao

**Affiliations:** 1 Guang'anmen Hospital, China Academy of Chinese Medical Sciences, Beijing 100053, China.; 2 Hubei University of Chinese Medicine, Hubei, Wuhan 430065, China.; 3 Beijing University of Chinese Medicine, Beijing 100029, China.; 4 The Chinese University of Hong Kong, Hong Kong SAR, China.; 5 Shuguang Hospital, Shanghai University of Traditional Chinese Medicine, Shanghai 201203, China.; 6 School of Medicine, Southern University of Science and Technology, Shenzhen, Guangdong, China.; 7 Department of Critical Care Medicine, Nanfang Hospital, Southern Medical University, Guangzhou 510515, China.; 8 Department of Cardiovascular Surgery, Sun Yat-sen Memorial Hospital, Sun Yat-sen University, Guangzhou 510120, China.

**Keywords:** DCM, DNA damage response, IFI16, fibrosis, ECM

## Abstract

**Rationale:** Dilated cardiomyopathy (DCM) is a severe cardiac condition characterized by ventricular dilation and systolic dysfunction, often leading to heart failure. While the DNA damage response (DDR) pathway is increasingly implicated in DCM pathogenesis, the precise mechanisms linking DDR activation to specific pathological features like adverse extracellular matrix (ECM) remodeling and fibrosis remain poorly understood. Interferon-inducible protein 16 (IFI16), a known DNA sensor involved in DDR and inflammatory signaling, emerges as a potential mediator in this process. This study aimed to investigate the role of the DDR-IFI16 axis in DCM, specifically exploring its connection to ECM dysregulation and cardiac dysfunction, and to evaluate its potential as a therapeutic target.

**Methods:** W This study integrated bioinformatics analyses of human cardiac transcriptomic datasets with experimental validation in a doxorubicin-induced murine DCM model. Cardiac function was assessed by echocardiography. Key molecular pathways were investigated using qPCR, ELISA, and enrichment analyses. Mechanistic roles were tested via pharmacological DDR inhibition *in vivo* and targeted *IFI16* siRNA knockdown *in vitro*, followed by analysis of fibrosis, cell viability, and cytotoxicity markers.

**Results:** Bioinformatic analyses consistently revealed activation of DDR and cytosolic DNA sensing pathways across human iPSC-CM models and *ex vivo* DCM heart tissue. WGCNA identified a key gene module strongly associated with DCM, co-enriched for DDR, DNA replication, and ECM/TGF-β signaling pathways. Single-cell RNA-seq analysis confirmed significant *IFI16* upregulation in human DCM samples. High *IFI16* expression strongly correlated with pathways governing 'Extracellular matrix organization' and key fibrotic genes. Experimental validation in the doxorubicin mouse model confirmed DDR activation. Crucially, *in vivo* treatment with the DDR inhibitor NU7441 significantly attenuated *IFI16* upregulation, ameliorated cardiac dysfunction, and decreased cardiac fibrosis markers. Complementarily, *in vitro* knockdown of *IFI16* significantly reduced pro-fibrotic markers, increased cell viability, and decreased cell injury.

**Conclusions:** Our findings delineate a novel pathogenic axis in DCM where nuclear stress-induced DDR activation drives the upregulation of the DNA sensor IFI16. IFI16 acts as a critical mediator linking DDR signaling to pathological ECM remodeling and fibrosis. Pharmacological inhibition of the upstream DDR pathway effectively mitigates IFI16 induction, attenuates cardiac fibrosis, and improves cardiac function. This study identifies the DDR-IFI16-ECM remodeling axis as a crucial contributor to DCM pathogenesis and highlights its potential as a therapeutic target for mitigating adverse cardiac remodeling and dysfunction.

## Introduction

The maintenance of genomic integrity is a fundamental requirement for cellular survival and proper tissue function. The DNA damage response (DDR) is a sophisticated and highly coordinated network that has evolved to detect, signal, and repair a wide array of DNA lesions, ensuring the faithful transmission of genetic information 1. When this intricate network is compromised, it can lead to genomic instability and contribute to the development of various human diseases 2. Interferon-inducible protein 16 (IFI16) is an intracellular sensor that recognizes both self and non-self DNA, playing a crucial role in the innate immune system, inflammation, and the regulation of cell death pathways. Dilated cardiomyopathy (DCM) is a condition characterized by the enlargement and weakening of the heart muscle, leading to impaired pumping ability and potentially heart failure, arrhythmias, and sudden cardiac death. The causes of DCM are diverse, encompassing genetic factors, viral infections, toxic exposures, and autoimmune responses.

The DNA damage response (DDR) is a fundamental cellular process that serves as a guardian of the genome, ensuring the integrity and accurate inheritance of genetic information 2. It is a complex and highly regulated network composed of numerous interconnected components that work in concert to detect, signal, and repair a vast array of DNA lesions 1. This intricate system is essential for maintaining cellular homeostasis and preventing the accumulation of genetic errors that can lead to disease. Emerging research suggests a complex interplay between DNA damage, inflammation, and the development of DCM 3. DNA damage can trigger inflammatory pathways, and chronic inflammation is known to contribute to the myocardial dysfunction observed in DCM.

Studies have shown that myocardial DNA damage is prevalent in DCM patients, with significant DNA strand breaks observed in cardiomyocytes 4. This damage is associated with interstitial fibrosis and a progressive loss of cardiac function. Besides, patients with pathogenic variants in sarcomere genes exhibit less DNA damage and a better treatment response compared to those with non-sarcomere gene variants 5. This suggests that DNA damage is a mediator between genotype and treatment outcomes in DCM 5. Mechanismically, endogenous DNA damage leads to oxidative stress and apoptosis in cardiac myocytes, contributing to DCM. The chronic activation of p53 and increased oxidative stress are key mechanisms driving this process 6. However, the key regulator links DDR and DCM have not been fully understood.

Interferon-inducible protein 16 (IFI16) is a key player in cellular responses to both pathogenic threats and cellular stress. IFI16 plays a crucial role in the DNA damage response and immune signaling pathways. It is a member of the HIN-200 family and acts as a DNA sensor, participating in various cellular processes, including the regulation of cell growth, apoptosis, and immune responses. IFI16's involvement in DNA damage response is particularly significant due to its interactions with key proteins such as BRCA1 and p53 7, 8, which are essential for maintaining genomic stability and initiating apoptosis in response to DNA damage. Additionally, FI16 is implicated in the activation of cell cycle checkpoints in response to DNA damage, as evidenced by its involvement in the phosphorylation of p53 and accumulation of p21WAF1, which are critical for cell cycle arrest 8. IFI16, with its capacity to sense DNA and activate inflammatory signaling, presents a potential molecular link between DNA damage and the pathogenesis of DCM 9. Furthermore, defects in the DDR system can lead to genomic instability and cellular stress, which may contribute to the deterioration of cardiac function in DCM 10. The convergence of these biological processes, all potentially influenced by IFI16, underscores the need for a comprehensive investigation into their interconnections. This report aims to explore the relationship between the DDR, IFI16, and DCM, focusing on IFI16's potential role as a mediator and marker, and to identify therapeutic implications arising from their interplay.

## Methods

### Mice

Procedures involving animals were carried out adhering to guidelines sanctioned by the Institutional Animal Care and Use Committee (IACUC) at China Academy of Chinese Medical Sciences. To establish a dilated cardiomyopathy (DCM) phenotype, C57BL/6 male rodents (20-25 g) were administered intraperitoneal (i.p.) doses of doxorubicin hydrochloride. The dosage was 5 mg/kg, given weekly across a six-week duration, utilizing a protocol adapted from prior research. Subjects in the control group received i.p. delivery of a matching volume of PBS following an identical timing regimen. For inhibiting the DNA Damage Response (DDR), certain mice received treatment with NU7441 (SelleckChem, Cat# S2638) 11, 12, recognized as a selective blocker for the DNA-dependent protein kinase catalytic subunit (DNA-PKcs). The inhibitor was prepared in a solution containing 10% DMSO to reach the target strength. This compound was given through the intraperitoneal route, utilizing a dosage of 3 mg/kg. Delivery of either NU7441 or its carrier solution started simultaneously alongside the initial doxorubicin dose and occurred each day throughout the entire doxorubicin regimen. Upon concluding the intervention phase, heart performance was evaluated using echocardiography 13, 14. Afterward, animals were humanely sacrificed for harvesting organ samples designated for subsequent examinations, including quantitative PCR and ELISA, to quantify markers related to DDR and fibrosis 15-18.

### Echocardiography

Evaluation of left ventricular (LV) metrics was carried out via echocardiography on non-anesthetized animals employing a GE Vivid 7 Dimension device coupled with a GE i13L 10-14 MHz probe. For examining LV diastolic performance, Doppler ultrasound techniques (pulse wave or tissue modes) were acquired using the Vevo3100 Imaging System (Fujifilm Visual Sonics) while subjects were under isoflurane anesthesia. Left ventricular measurements were subsequently calculated as the mean value derived from three sequential cardiac cycles 19, 20.

### Cell culture

The HL-1 cardiomyocyte line was propagated using a specialized Claycomb formulation (Sigma-Aldrich), fortified with 10% fetal bovine serum, penicillin (100 U/ml), streptomycin (100 μg/ml), norepinephrine (0.1 mM), plus L-glutamine (2 mM). Growth surfaces were pre-treated with a combination containing fibronectin and gelatin (both Sigma-Aldrich) to enhance cellular attachment. Propagation occurred within an incubator providing humidity, set to 37 °C and supplying 5 % carbon dioxide, offering ideal conditions for proliferation and viability 21, 22. For experimental procedures, cardiomyocytes were plated into standard 6-well formats using an initial cell number calculated to achieve 70-80 % surface coverage when interventions began. Upon reaching the target density, cultures were exposed to doxorubicin (Sigma-Aldrich) at a 5 μm/L. This agent was derived from a concentrated stock dissolved in either sterile H₂O or phosphate-buffered saline (PBS) and introduced straight to the growth media, ensuring the carrier solvent level stayed under 0.1 %. Parallel control cultures were administered a matching amount of the carrier solution alone 23, 24. This exposure period lasted for two days within the normal incubator environment. Subsequently, cellular material was collected for subsequent investigations aimed at evaluating indicators and characteristics linked to the dilated cardiomyopathy condition 25, 26.

### Total RNA isolation and real-time PCR analysis

Cellular ribonucleic acid extraction was accomplished employing the TransZol Up Plus RNA Kit (TransGen Biotech, Beijing, China; Cat# ER501-01). The extracted RNA subsequently served as a template for conversion into complementary DNA (cDNA) utilizing random hexamers alongside SuperScript III reverse transcriptase (Thermo Fisher Scientific, Waltham, MA, USA; Cat# 12574035). Quantitative PCR (qPCR) analysis was conducted on an ABI 7900HT platform (Life Technologies) using reaction mixtures containing TransStart® Top Green qPCR SuperMix (TransGen Biotech, Beijing, China; Cat# AQ131-01) 27, 28. Cycling parameters applied during qPCR runs involved an initial 10 min hold at 95 °C for enzyme activation, succeeded by 40 amplification rounds comprised of: denaturing at 95 °C for 30 s, annealing at 60 °C for 30 s, and extension at 72 °C for 30 s. The presence of solitary peaks upon dissociation curve assessment verified amplification product specificity 29, 30. 18S ribosomal RNA served as the internal reference for transcript quantification, and relative expression changes were determined employing the 2^-ΔΔCt^ approach for comparisons 31, 32. Oligonucleotides targeting specific genes utilized in the qPCR assays included: Bax, Fwd: 5'-TGAAGACAGGGGCCTTTTTG-3', Rev: 5'-AATTCGCCGGAGACACTCG-3'; Ogg1, Fwd: 5'-CTGCCTAGCAGCATGAGACAT-3', Rev: 5'-CAGTGTCCATACTTGATCTGCC-3'; Xpc, Fwd: 5'-TCCAGGGGACCCCACAAAT-3', Rev: 5'-GCTTTTTGGGTGTTTCTTTGCC-3'; Ku70, Fwd: 5'-ATGTCAGAGTGGGAGTCCTAC-3', Rev: 5'-TCGCTGCTTATGATCTTACTGGT-3'; Chk2, Fwd: 5'-TGACAGTGCTTCCTGTTCACA-3', Rev: 5'-GAGCTGGACGAACCCTGATA-3'; Col1a1, Fwd: 5'-GCTCCTCTTAGGGGCCACT-3', Rev: 5'-CCACGTCTCACCATTGGGG-3'; β-actin: Fwd: 5'-TCTGGCACCACACCTTCTA-3', Rev: 5'-AGGCATACAGGGACAGCAC-3'.

### siRNA transfection

For *in vitro* gene silencing studies, HL-1 cells were cultured in Dulbecco's Modified Eagle Medium (DMEM) supplemented with 10 % fetal bovine serum (FBS), 100 U/mL penicillin, and 100 µg/mL streptomycin and maintained in a humidified incubator at 37 °C with 5 % CO_2_. Cells were seeded into 6-well plates at a density of 1 x 10^5^ cells/well for 6-well plates and allowed to adhere for approximately 24 h. Subsequently, cells were transfected with either a validated small interfering RNA targeting *IFI16* (si-IFI16) or a non-targeting control siRNA (si-Ctrl) at a final concentration of 25 nM. Transfection was performed using Lipofectamine RNAiMAX Transfection Reagent (Invitrogen) following the manufacturer's instructions 33-35. Briefly, siRNA and the transfection reagent were individually diluted in Opti-MEM I Reduced Serum Medium (Gibco), then combined and incubated for 10-20 minutes at room temperature to allow complex formation before being added dropwise to the cells in fresh culture medium. After 6 h of incubation, the transfection medium was replaced with fresh complete growth medium. At 72 h post-transfection, cells were collected for mRNA analysis (*Col1a1*, *Col1a3*), or cell culture supernatants were collected for TGF-β activity measurement, or cells were subjected to MTT assays for viability assessment and LDH release assays for cytotoxicity measurement according to standard protocols 36, 37.

### ELISA assays

Following enumeration, cells were dispensed into 96-well format dishes and subsequently incubated for one day. After this incubation period, conditioned media was harvested 38, 39. Passage through 0.45 μm filters then occurred in order to remove any non-adherent cells or debris. Levels of both Transforming Growth Factor-beta (TGF-β) and gamma-H2AX protein present within the collected media were quantified employing dedicated enzyme-linked immunosorbent assay kits sourced from Abcam (Cambridge, UK). Every specimen was assessed using multiple independent biological preparations, with triplicate measurements performed for each preparation 40, 41.

### MTT and LDH assays

HL-1 cardiomyocytes underwent cultivation followed by exposure to doxorubicin at 5 μm/L concentration over a two-day period, creating an *in vitro* system mimicking aspects of dilated cardiomyopathy (DCM) 42, 43. To evaluate metabolic activity, after the drug exposure duration, the MTT compound was introduced straight into the well contents, reaching a 0.5 mg/ml final concentration. Incubation proceeded at 37 °C for three to 4 h, allowing violet formazan precipitate formation. Afterwards, the overlaying liquid was aspirated, and the precipitate was dissolved using dimethyl sulfoxide (DMSO); optical density readings taken at 570 nm via a plate spectrophotometer served for determining relative cell survival 44, 45.

Concurrently, cytotoxicity assessment via LDH release was conducted by gathering the conditioned media following the same drug exposure period. Extracellular lactate dehydrogenase activity was measured utilizing a purchased LDH assay kit per the supplier's guidelines. This involved combining the gathered media with the kit's reagent solution (containing NADH and a tetrazolium salt); generation of the colored formazan derivative was then assessed via absorbance reading at 490 nanometers. Untreated control groups were administered a matching amount of the carrier solution. Each assay condition was performed three times independently for robust data analysis 46, 47.

### Raw data processing

This investigation utilized public repositories on Gene Expression Omnibus (GEO) under accession numbers GSE5406 and GSE197850. The GSE197850 dataset comprised samples from 4 cardiomyocytes representing dilated models alongside 4 corresponding control heart cells. Meanwhile, dataset GSE5406 contained eighty-six heart tissue specimens derived from DCM patients plus sixteen specimens from non-diseased hearts 48, 49. Information regarding genes associated with DNA damage responses (DARGs) was sourced through the GeneCards resource 50, 51.

### Data filtering and processing

Precise transcript abundance information was obtained through custom Perl scripts implementing alignment and ordering procedures on the gene expression datasets. Subsequently, a normalization step was performed on both the GSE5406 and GSE197850 cohorts 52, 53. Specifically, the "limma" package within R software handled the pre-processing plus normalization tasks for the initial gene count tables derived from the aforementioned GEO repositories 54-57.

### Co-expression gene identification and enrichment analyses

Application of the Weighted Gene Co-expression Network Analysis (WGCNA) approach enabled gene grouping into modules, and subsequently, relationships connecting these gene groups with specific characteristics were evaluated 58, 59. Network structures based on co-expression were built employing the upper quartile of genes exhibiting the highest variance within the GSE5406 cohort data. The dynamic tree cutting technique, utilizing a merging threshold set at 0.25, facilitated the consolidation of modules. Subsequently, modules displaying the highest correlation with relevant traits were identified and characterized. Functional enrichment investigations were performed utilizing the R software package 'clusterProfiler'. Furthermore, Gene Set Enrichment Analysis (GSEA) was employed to discover relevant biological processes or routes.

### ScRNA seq analysis

The single-cell RNA sequencing (scRNA-seq) dataset GSE121893 was acquired via the Gene Expression Omnibus (GEO) repository. Subsequent analysis of this scRNA-seq data was carried out in the R environment, utilizing primarily version 4.3.0.1 of the Seurat toolkit for sample processing. Initial quality control involved removing 12 individual cells exhibiting feature counts below 200 or exceeding 5,000 detected genes 60-62. Following library size normalization (accomplished via NormalizeData), the two thousand features showing greatest variability were selected employing the FindVariableFeatures routine. Expression values for these variable features were subsequently scaled utilizing the ScaleData command. Principal component analysis (PCA) dimensionality reduction was conducted based on the scaled variable genes (executed via RunPCA), aiming to condense the feature space within the single-cell dataset. The Harmony algorithm implementation in R served for integrating batch effects across the eighteen distinct samples 63, 64. Uniform Manifold Approximation and Projection (UMAP) visualizations were produced based on the Harmony-corrected dimensions (computed with RunUMAP). Correspondingly, cell populations were grouped via a graph clustering approach utilizing the Harmony-integrated space, applying a resolution parameter of 0.7. Employment of the FindAllMarkers command allowed identification of distinguishing transcripts for every cell group. Subsequently, population types were assigned by consulting published cell type signatures alongside the CellMarker version 2.0 resource. Comparisons of gene expression profiles between specified populations were performed via the FindMarkers tool, utilizing the Wilcoxon rank-sum statistical test. The Benjamini-Hochberg procedure was applied for false discovery rate adjustment on the results 65, 66.

### Statistics

Assessment of data normality employed the Shapiro-Wilk procedure. Evaluating differences between two independent groups involved an unpaired, two-sided Student's t-test, incorporating Welch's adjustment if variances were found to be unequal. Alternatively, the Mann-Whitney U test was applied when comparing two sets of data lacking a normal distribution pattern. When assessing differences across three or more groups, appropriate analysis of variance (one-way, two-way, or two-way repeated measures) was utilized, succeeded by post-hoc analysis using Sidak's correction for multiple comparisons. For non-parametric multi-group comparisons (if any group deviated from normality), the Kruskal-Wallis H test was employed, followed by Dunn's post-hoc test for pairwise assessments 67, 68. Results are presented showing the mean value plus or minus the standard error of the mean (SEM). A threshold for statistical significance was established at a P-value below 0.05. All statistical computations were executed using GraphPad Prism software, version 10.

## Results

### Activation of DNA damage sensing and inflammatory pathways in an iPSC-CM model of dilated cardiomyopathy

**Figure 1:**
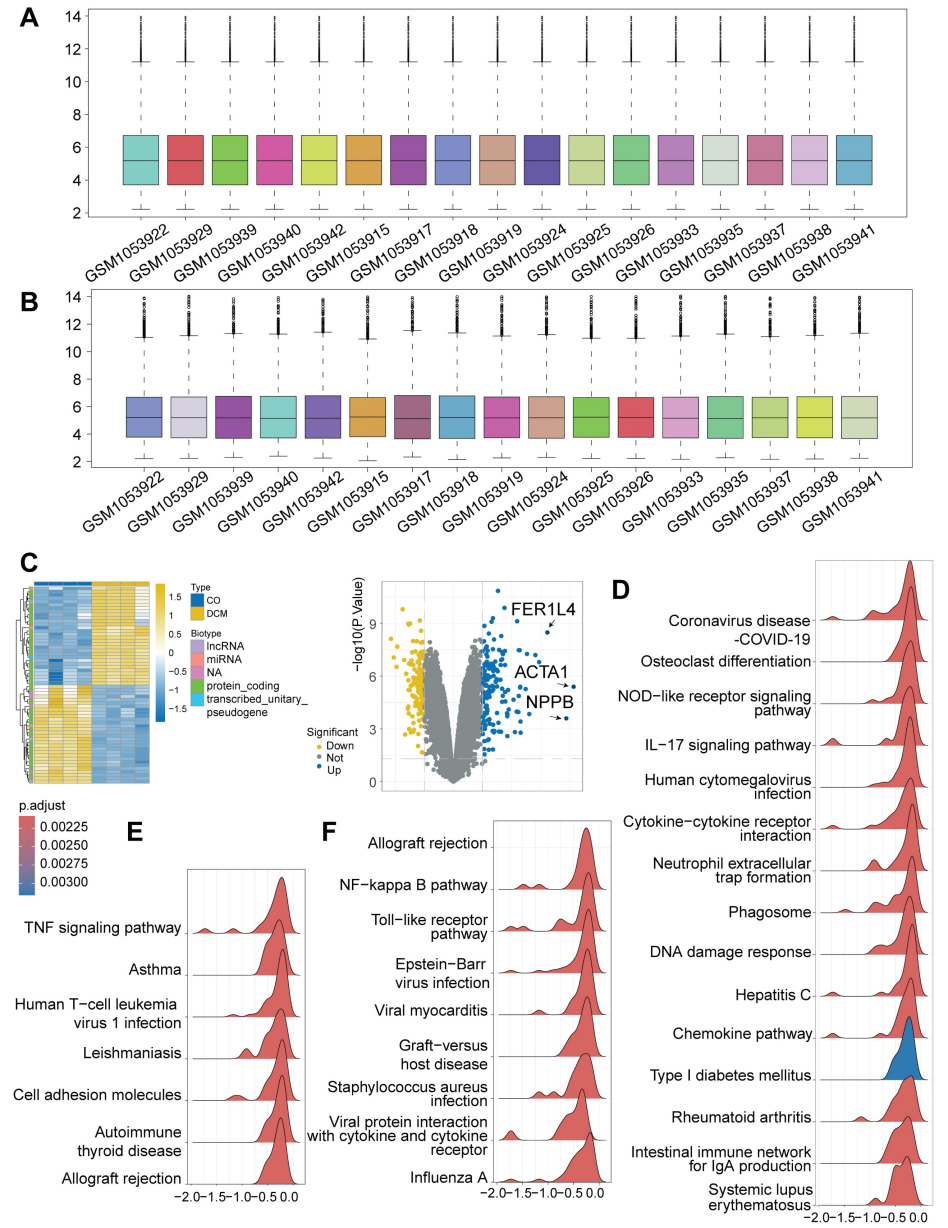
** Bioinformatic analysis of RNA-seq data from GSE197850 reveals activation of DNA damage sensing and inflammatory pathways in a human iPSC-CM model of dilated cardiomyopathy (DCM). (A, B)** Box plots illustrating the distribution of expression values for each sample from the GSE197850 dataset before (A) and after (B) normalization. Each box represents the interquartile range (IQR), with the central line indicating the median. Whiskers extend to 1.5 times the IQR. Normalization aligned median expression levels across samples. **(C)** Differential gene expression analysis between the DCM model group and control induced pluripotent stem cell-derived cardiomyocytes (iPSC-CMs). **(D, E, F)** Gene Set Enrichment Analysis (GSEA) plots showing pathways significantly enriched in the DCM model group compared to controls. Plots illustrate the enrichment score (ES) reflecting the degree to which a gene set is overrepresented at the extremes of the ranked gene list. Representative enriched pathways are shown, including (D) "Cytosolic DNA-sensing" and inflammatory pathways, (E) additional immune/inflammatory pathways like "TNF signaling", and (F) pathways such as "NF-kappa B signaling" and "Toll-like receptor signaling". Peaks indicate enrichment of the respective gene set.

To investigate the transcriptional landscape associated with cardiomyopathy and the potential activation of DNA Damage Response (DDR), we analyzed high-throughput RNA-sequencing data from the publicly available GSE197850 dataset. This dataset profiled human induced pluripotent stem cell-derived cardiomyocytes (iPSC-CMs), providing an *in vitro* model system. Our analysis specifically compared the transcriptomes of the dilated cardiomyopathy (DCM) model group (n = 4) against the control iPSC-CM group (n = 4) within this dataset. Following data preprocessing and normalization procedures to ensure robust inter-sample comparability (Figure 1A-B), differential expression analysis was performed. This identified a substantial number of differentially expressed genes (DEGs) distinguishing the DCM model iPSC-CMs from the controls, revealing a distinct disease-associated transcriptional signature visualized by heatmap clustering and volcano plot analysis (Figure 1C), including significant alterations in genes like *ACTA1* and *NPPB*. To elucidate the functional implications of these transcriptional changes, Gene Set Enrichment Analysis (GSEA) was conducted. Notably, this analysis demonstrated a significant enrichment of the "Cytosolic DNA-sensing" pathway within the DCM model group (Figure 1D), strongly suggesting the activation of DDR mechanisms in these cardiomyocytes. Furthermore, consistent with a potential interplay between cellular stress and inflammation, pathways related to immune activation, such as "NF-kappa B signaling," "TNF signaling," "Toll-like receptor signaling," and "Cytokine-cytokine receptor interaction," were also found to be significantly enriched (Figure 1D-F). Taken together, these bioinformatic analyses of iPSC-CM provide compelling evidence for significant transcriptomic alterations in this *in vitro* model of DCM, characterized by the pronounced activation of pathways involved in DNA damage sensing and pro-inflammatory signaling.

### Enrichment of DNA replication, repair, and cell cycle pathways in the iPSC-CM DCM model

**Figure 2:**
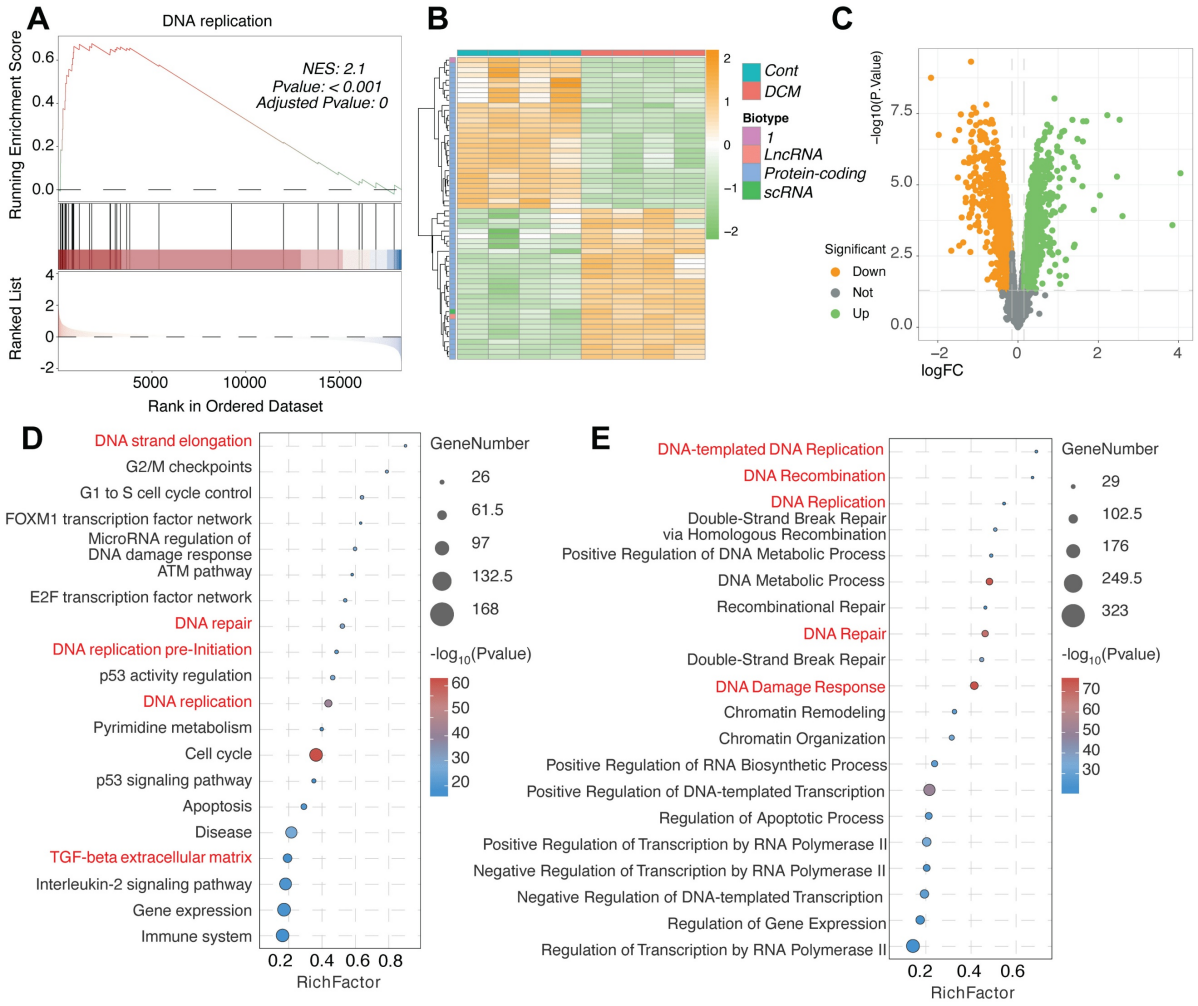
** Enrichment of DNA replication, DNA damage response, and cell cycle pathways in the iPSC-CM model of dilated cardiomyopathy from GSE197850. (A)** Gene Set Enrichment Analysis (GSEA) plot showing significant enrichment of the "DNA replication" pathway in the DCM model group compared to control induced pluripotent stem cell-derived cardiomyocytes (iPSC-CMs). The plot displays the enrichment score (ES) profile, the positions of gene set members on the ranked list of genes, and the corresponding Normalized Enrichment Score (NES), P-value, and Adjusted P-value. **(B)** Heatmap illustrating the expression patterns of selected genes, including lncRNAs, protein-coding genes, and scRNAs potentially involved in DNA damage response (DDR) and replication pathways. Rows represent genes, columns represent individual samples from Control (Cont) and DCM groups. Expression levels are indicated by color intensity, and data are clustered hierarchically. **(C)** Volcano plot visualizing differentially expressed genes (DEGs) between the DCM model and control iPSC-CMs. Points are colored based on significance thresholds. **(D, E)** Bubble plots summarizing pathway enrichment analysis results for DEGs identified between DCM and control groups. The x-axis indicates the Rich Factor, bubble size corresponds to the number of DEGs in the pathway, and bubble color represents the statistical significance. Key enriched pathways related to (D) DNA strand elongation, cell cycle checkpoints (G2/M, G1 to S), DNA replication pre-initiation, ATM pathway, p53 regulation, and beta regulation of extracellular matrix, and (E) DNA-templated DNA replication, DNA recombination, double-strand break repair, DNA damage response, and chromatin remodeling are shown.

Further investigation into the molecular consequences of DNA Damage Response (DDR) activation in the DCM model iPSC-CMs from the GSE197850 dataset revealed significant alterations in related cellular processes. GSEA demonstrated a highly significant positive enrichment for the "DNA replication" pathway in the DCM group compared to controls (Figure 2A), suggesting an aberrant activation of DNA synthesis machinery. This finding was supported by the differential expression patterns of specific protein-coding and non-coding genes potentially involved in these pathways, as visualized by heatmap clustering which distinguished DCM samples from controls (Figure 2B), and further illustrated in the volcano plot highlighting numerous significantly altered genes (Figure 2C). Comprehensive pathway enrichment analyses provided additional detail, revealing significant overrepresentation of gene sets associated not only with "DNA replication" and "DNA repair" (including specific mechanisms like "Double-Strand Break Repair via Homologous Recombination"), but also intimately linked processes such as "DNA Damage Response," cell cycle control (e.g., "G2/M checkpoints," "G1 to S cell cycle control," "Cell cycle"), and key regulatory pathways like the "ATM pathway" and "p53 activity regulation" (Figure 2D-E). Interestingly, enrichment related to "Beta regulation of extracellular matrix" was also detected (Figure 2D), potentially linking the observed DDR to extracellular remodeling processes relevant to DCM. Taken together, these results indicate a complex molecular phenotype in the DCM model iPSC-CMs characterized by the concurrent upregulation of DNA replication, DNA repair, and cell cycle control pathways alongside DDR activation.

### DDR activation and suppressed DNA replication characterize human DCM hearts

**Figure 3:**
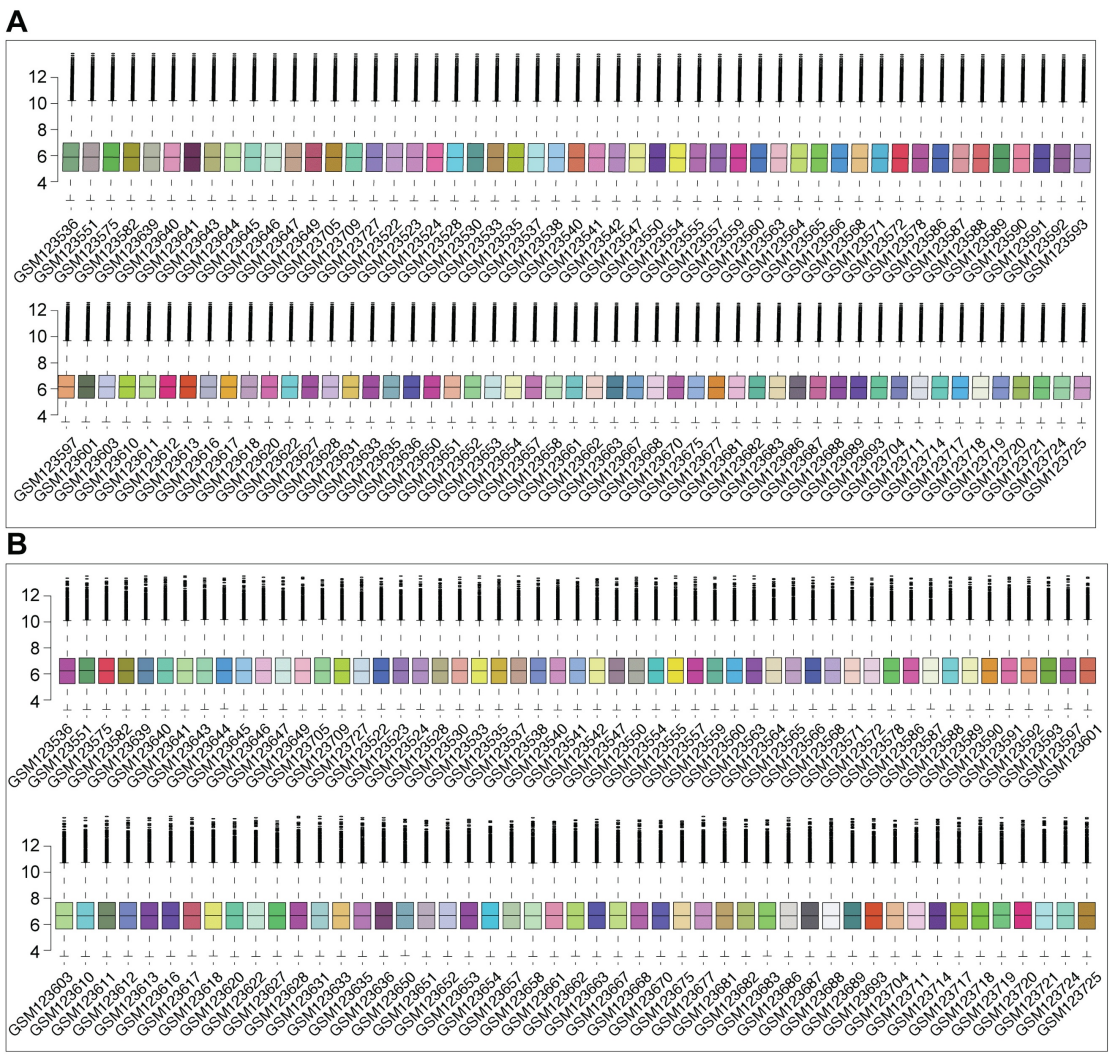

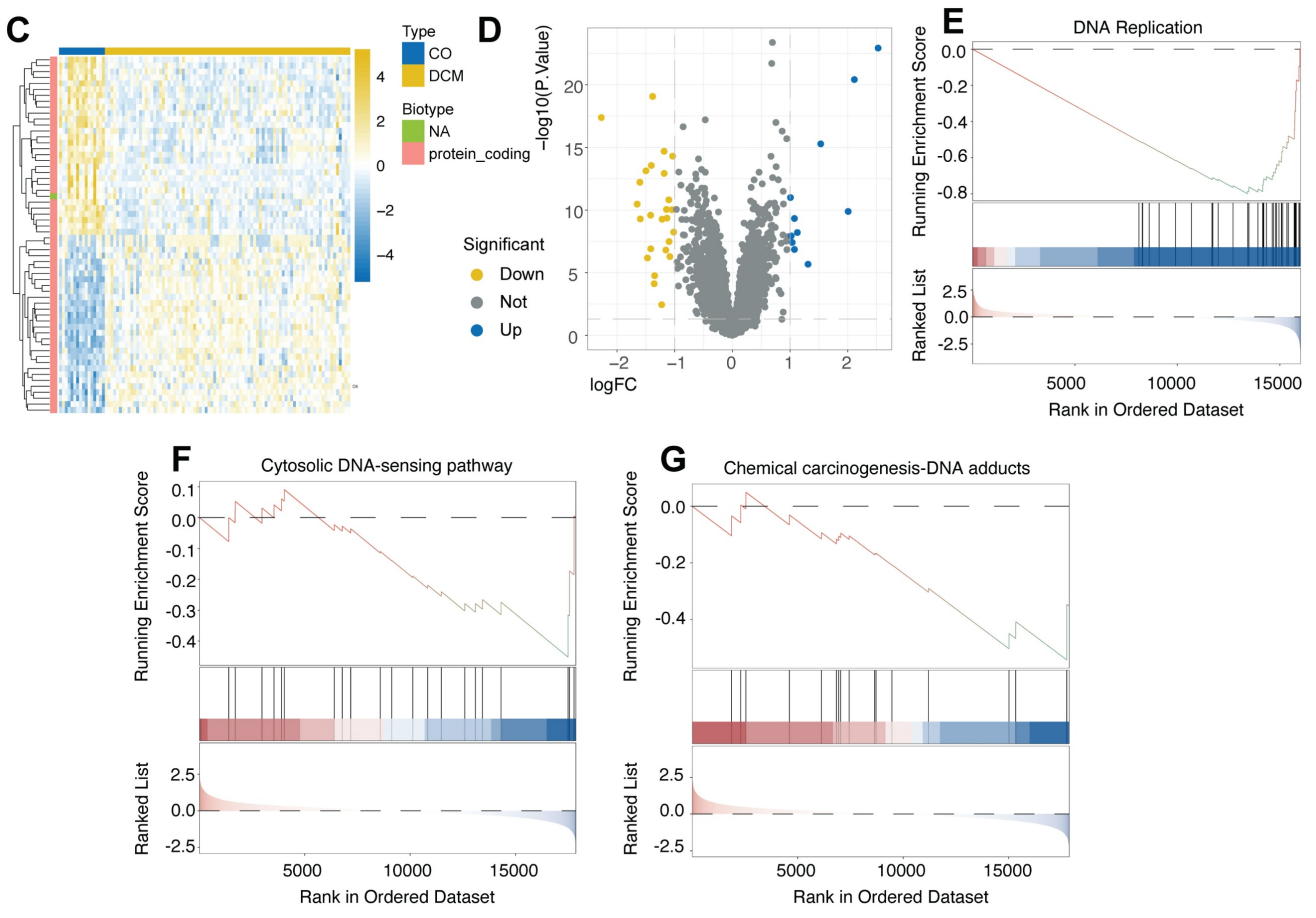
** Activation of DNA damage sensing pathways and suppression of DNA replication revealed by analysis of human heart tissue transcriptomes from GSE5406. (A, B)** Box plots depicting the distribution of expression values from Affymetrix microarray data for each human left ventricular myocardium sample in the GSE5406 dataset, shown before (A) and after (B) data normalization. Normalization ensured comparability across arrays. **(C)** Heatmap displaying hierarchical clustering of samples (columns) and significant differentially expressed genes (DEGs) (rows) identified between dilated cardiomyopathy (DCM) and non-failing (NF) control tissues. Sample group annotation and gene biotypes are indicated. **(D)** Volcano plot illustrating DEGs between DCM and NF groups. **(E, F, G)** Gene Set Enrichment Analysis (GSEA) plots comparing DCM versus NF control tissues. GSEA revealed (E) significant negative enrichment (downregulation) of the "DNA Replication" pathway, (F) significant positive enrichment (activation) of the "Cytosolic DNA-sensing pathway", and (G) significant positive enrichment of the "Chemical carcinogenesis - DNA adducts" pathway in the DCM samples.

To further validate the activation of DDR in human DCM using *ex vivo* tissue, we analyzed the publicly available GSE5406 dataset. This dataset comprises expression profiles generated using Affymetrix Human Genome U133A microarrays on human left ventricular myocardium. Samples were obtained during cardiac transplantation from patients with advanced DCM (including idiopathic and ischemic etiologies) and from non-failing (NF) donor hearts used as controls. Following quality control and array data normalization procedures to ensure comparability across samples (Figure 3A-B), differential expression analysis was performed comparing DCM tissues to NF controls. This analysis identified a substantial number of DEGs that effectively distinguished the DCM samples from the NF group, as demonstrated by hierarchical clustering (Figure 3C) and volcano plot visualization (Figure 3D), indicating significant transcriptomic remodeling in the diseased human heart. Subsequent GSEA revealed key perturbed biological pathways. Notably, significant positive enrichment of the "Cytosolic DNA-sensing pathway" (Figure 3F) and the "Chemical carcinogenesis-DNA adducts" pathway (Figure 3G) was observed in DCM tissues relative to controls, providing strong evidence for DDR activation and response to DNA damage in the failing human myocardium. Conversely, GSEA revealed a significant negative enrichment of the "DNA Replication" pathway (Figure 3E) in DCM hearts, suggesting a suppression of DNA synthesis pathways in this *ex vivo* pathological context. Therefore, analysis of the human heart tissue microarray dataset confirms the activation of DNA damage sensing pathways in advanced human DCM, which occurs concomitantly with a downregulation of DNA replication processes.

### WGCNA identifies a key co-expression module linking DDR and ECM/fibrosis pathways in DCM

**Figure 4:**
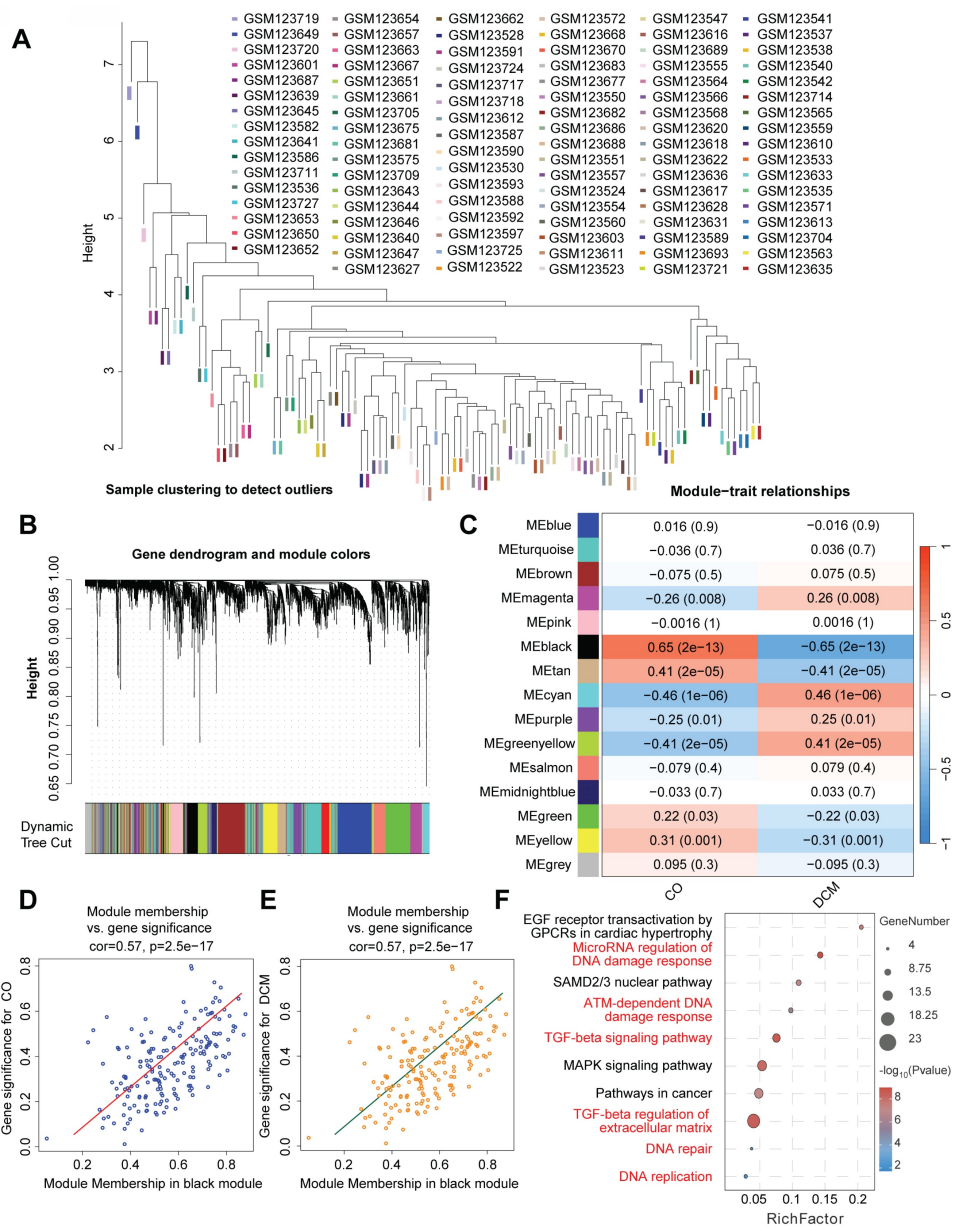
** Weighted gene co-expression network analysis (WGCNA) identifies modules associated with dilated cardiomyopathy enriched in DNA damage response and extracellular matrix pathways. (A)** Sample clustering dendrogram based on gene expression profiles used for WGCNA, facilitating the identification of potential outliers. Samples are indicated by their GSM IDs. **(B)** Hierarchical clustering dendrogram of genes based on topological overlap matrix (TOM) dissimilarity. Branches of the dendrogram represent clusters of highly co-expressed genes. Below the dendrogram, colors indicate distinct gene co-expression modules identified using the dynamic tree cut algorithm. **(C)** Heatmap illustrating the correlation between module eigengenes (MEs) and clinical traits (DCM/IHF vs. Control). Each cell represents the correlation coefficient and the corresponding P-value for a specific module-trait relationship. **(D, E)** Scatter plots showing the relationship between Gene Significance (GS) and Module Membership (MM) within the black module. (D) GS for control status versus MM in the black module. (E) GS for DCM status versus MM in the black module. Correlation coefficients (cor) and P-values are indicated for each plot. **(F)** Bubble plot displaying results of pathway enrichment analysis performed on genes within the black module. The x-axis represents the Rich Factor, bubble size indicates the number of genes enriched in the pathway, and color intensity corresponds to the significance. Key enriched pathways related to DNA damage response, DNA replication, TGF-beta signaling, and extracellular matrix regulation are shown.

To identify networks of co-expressed genes associated with DCM, specify which term is appropriate for your samples] and gain insights into their biological roles, we performed Weighted Gene Co-expression Network Analysis (WGCNA) on the transcriptomic data comparing diseased samples with normal controls. Initial sample clustering confirmed data quality and identified potential outliers (Figure 4A). The WGCNA algorithm identified 15 distinct modules of co-expressed genes, delineated by unique colors following hierarchical clustering and dynamic tree cutting (Figure 4B). Correlating module eigengenes (MEs), which represent the overall expression profile of each module, with clinical traits revealed several modules significantly associated with the disease state (Figure 4C). Notably, the black module demonstrated the strongest positive correlation with DCM/IHF status, while the turquoise and cyan modules were significantly negatively correlated. Further investigation of the highly significant black module showed a strong positive correlation between Module Membership (MM) and Gene Significance (GS) for DCM (Figure 4D-E), indicating that genes most central to the black module network are also highly associated with the disease phenotype (Figure 4D-E). Functional enrichment analysis of genes within the black module identified significant overrepresentation of pathways related to DNA Damage Response (DDR), including "DNA damage response," "ATM-dependent DNA damage response," and "DNA repair," as well as "DNA replication" (Figure 4F). Importantly, pathways crucial for extracellular matrix (ECM) remodeling and fibrosis, namely the "TGF-beta signaling pathway" and "TGF-beta regulation of extracellular matrix," were also significantly enriched within this module. Taken together, these WGCNA findings highlight specific gene co-expression networks correlated with DCM, with the most significantly associated module (black) integrating genes involved in both DDR/DNA replication processes and pathways governing ECM remodeling and fibrosis.

### Upregulation of DNA damage response markers in experimental doxorubicin-induced cardiomyopathy

**Figure 5:**
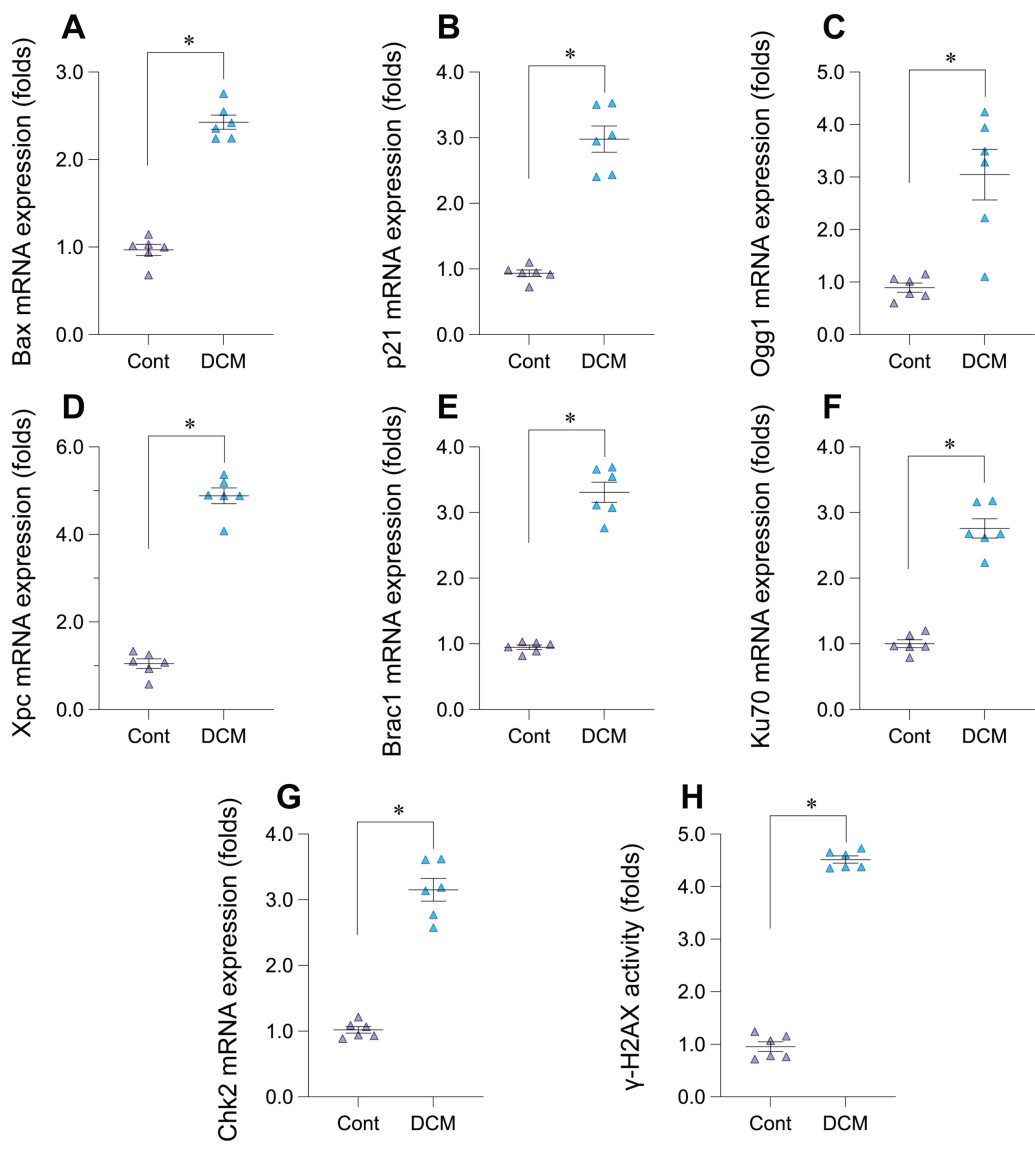
** Experimental validation of DNA damage response (DDR) pathway activation in a doxorubicin-induced mouse model of dilated cardiomyopathy (DCM). (A-G)** Relative mRNA expression levels of DDR-related genes in cardiac tissue from control (Cont) and doxorubicin-induced DCM mice, determined by quantitative PCR (qPCR). Genes measured include (A) *Bax*, (B) *p21*, (C) *Ogg1*, (D) *Xpc*, (E) *Brca1*, (F) *Ku70*, and (G) *Chk2*. Data are presented as fold change relative to the control group. **(H)** Relative activity levels of phosphorylated histone H2AX (γ-H2AX) in cardiac tissue from Control (Cont) and DCM mice Data are presented as fold change relative to the control group. For all panels, individual data points represent biological replicates (individual animals). Data are shown as mean ±SEM. *p < 0.05 vs Control group.

To experimentally validate the bioinformatic findings suggesting DNA Damage Response (DDR) activation in Dilated Cardiomyopathy (DCM), we employed a murine model induced by chronic doxorubicin administration (5 mg/kg weekly, 6 weeks) and compared results to saline-treated controls. Analysis of cardiac tissue from this model revealed significant alterations in key DDR markers. A primary indicator of DNA double-strand breaks, phosphorylated histone H2AX (γ-H2AX), showed significantly increased activity levels in the DCM group compared to controls (Figure 5H). Consistent with checkpoint activation, the mRNA expression of checkpoint kinase 2 (*Chk2*) was significantly upregulated (Figure 5G). Furthermore, components of multiple DNA repair pathways exhibited increased mRNA expression in DCM hearts, including *Ogg1* (base excision repair, Figure 5C), *Xpc* (nucleotide excision repair, Figure 5D), *Brca1* (homologous recombination, Figure 5E), and *Ku70* (non-homologous end joining, Figure 5F). We also observed significant upregulation of downstream DDR effectors, including the p53-target genes *p21*, involved in cell cycle arrest (Figure 5B), and the pro-apoptotic gene *Bax* (Figure 5A). Collectively, these results from the doxorubicin-induced DCM model demonstrate significant activation across multiple facets of the DDR pathway - including damage sensing, checkpoint signaling, DNA repair mechanisms, and downstream effectors - providing strong experimental validation for DDR activation in this cardiomyopathy context.

### Single-cell RNA-seq analysis pipeline reveals cellular heterogeneity in cardiac samples

**Figure 6:**
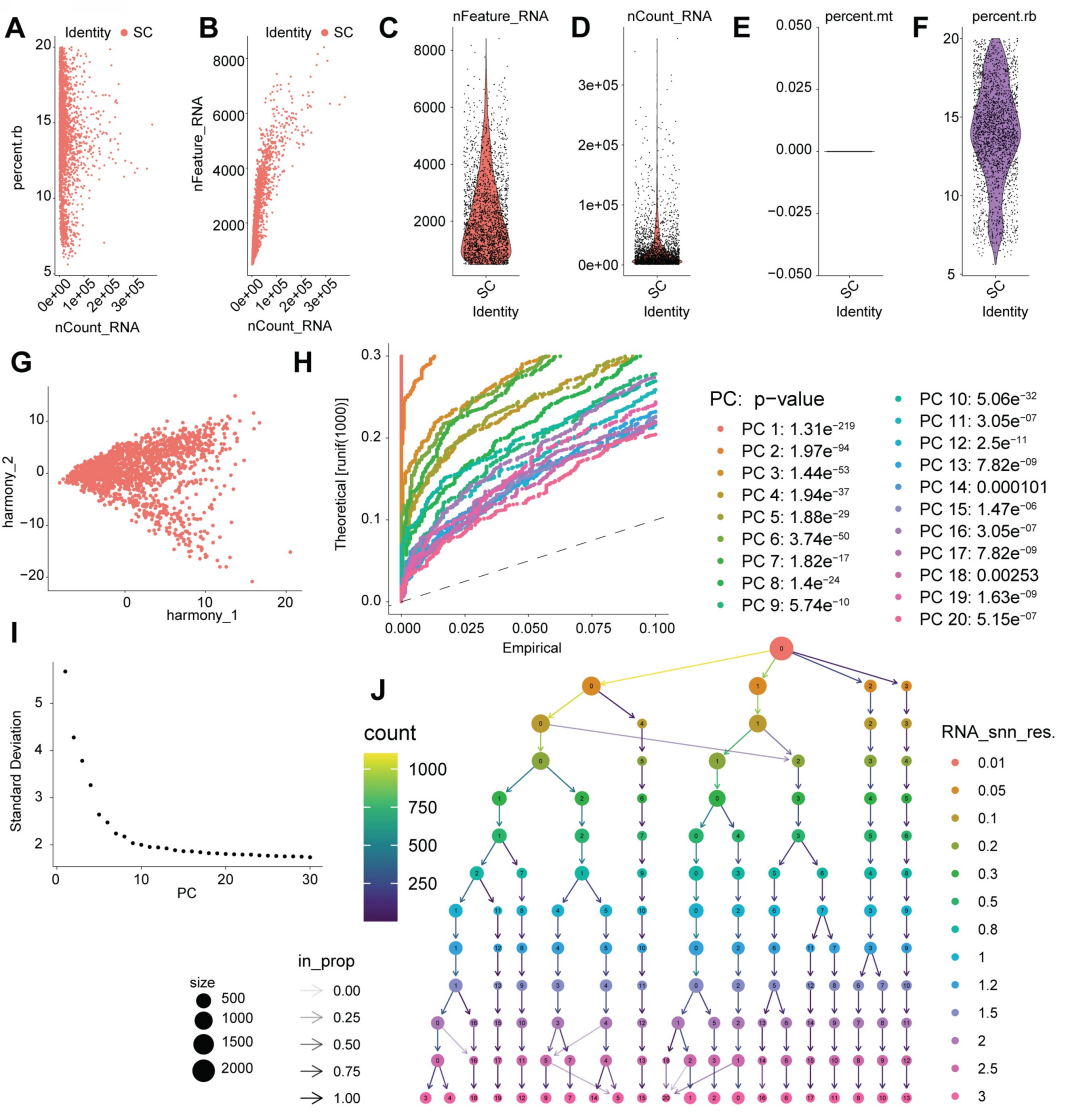
** Processing, quality control, and clustering of single-cell RNA sequencing data. (A-F)** Quality control (QC) metrics for the scRNA-seq dataset. (A, B) Scatter plots showing the relationship between the number of detected genes (nFeature_RNA), total transcript counts (nCount_RNA), and percentage of ribosomal reads (percent.rb) per cell. (C, D, F) Violin plots illustrating the distribution of nFeature_RNA, nCount_RNA, and percent.rb across cells. (E) Visualization of the percentage of mitochondrial reads (percent.mt). These metrics were used to filter out low-quality cells and potential doublets. **(G)** Scatter plot displaying cells projected onto Harmony coordinates, illustrating the results of data integration to correct for potential batch effects. **(H)** Quantile-Quantile (QQ) plot comparing the distribution of P-values for principal components (PCs) against the uniform distribution, assessing PC significance. Significant PCs deviate from the diagonal line. Listed P-values indicate the significance of the top 20 PCs. **(I)** Elbow plot showing the standard deviation associated with each principal component (PC), used to determine the optimal number of PCs for downstream dimensionality reduction and clustering. **(J)** Visualization of unsupervised clustering results based on significant PCs. Node size corresponds to the number of cells in the cluster, and node color represents cluster identity.

To complement insights gained from bulk transcriptomic analyses (GSE197850 iPSC-CM RNA-seq and GSE5406 human heart tissue microarrays) and to investigate cellular heterogeneity in DCM, we performed single-cell RNA sequencing (scRNA-seq) analysis. The data processing workflow is outlined in Figure 6. Initial quality control involved filtering cells based on standard metrics, including the number of detected genes (nFeature_RNA), transcript counts (nCount_RNA), and percentage of mitochondrial/ribosomal reads (percent.mt/percent.rb), ensuring the removal of low-quality cells or potential doublets (Figure 6A-F). To address potential batch effects, data integration was performed using Harmony] (Figure 6G). Principal Component Analysis (PCA) was applied for dimensionality reduction, and significant principal components (PCs) capturing meaningful biological variation were selected based on statistical evaluation and variance contribution (Figure 6H-I). Utilizing these significant PCs, unsupervised graph-based clustering identified distinct cell populations within the cardiac cellular milieu (Figure 6J). This comprehensive scRNA-seq processing pipeline yielded well-defined cell clusters, providing a necessary foundation for detailed downstream analyses at single-cell resolution, such as examining cell-type-specific expression patterns of genes like *IFI16* in the context of DCM.

### Single-cell analysis identifies cardiac cell populations with enriched DDR/ECM pathways relevant to IFI16 activity

**Figure 7:**
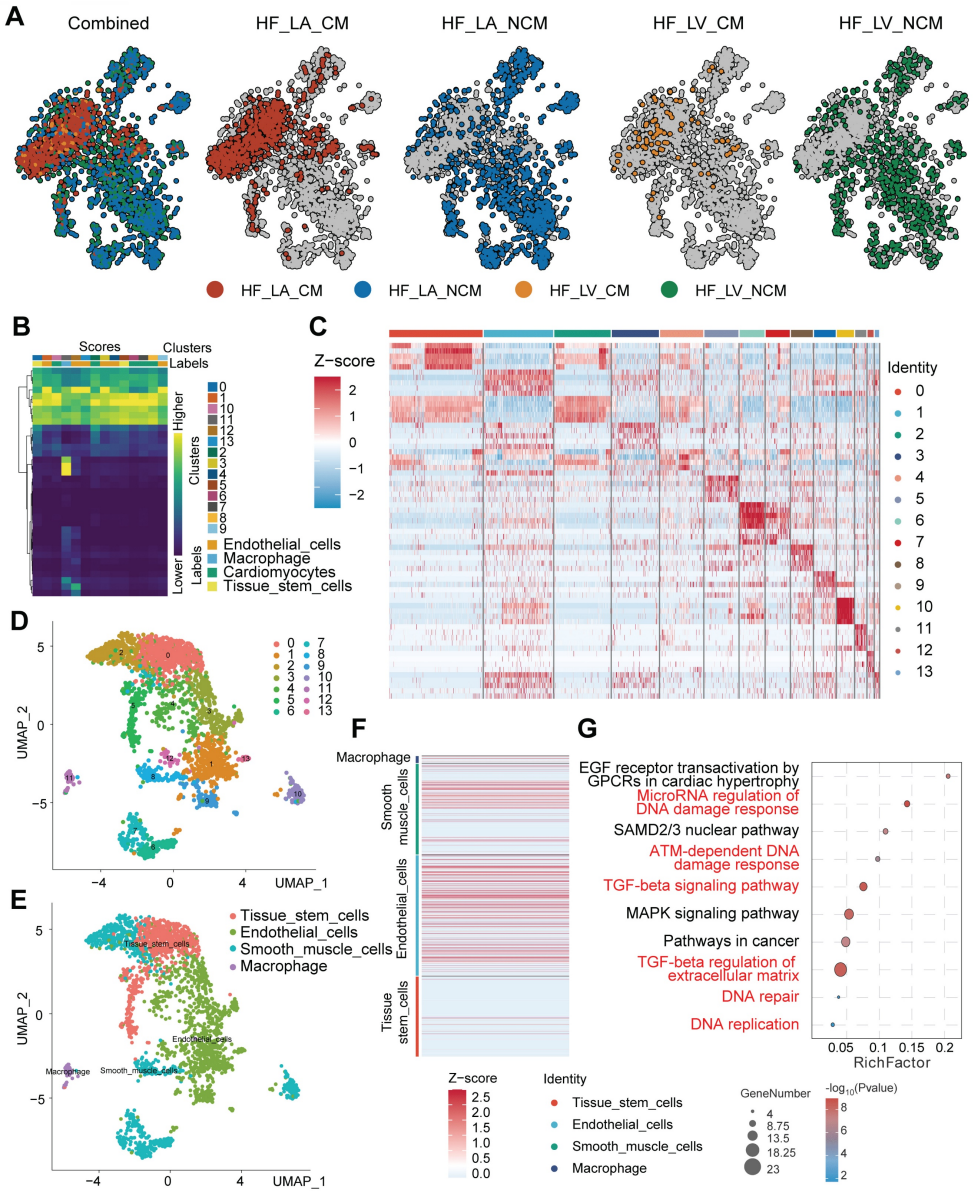
** Single-cell RNA sequencing analysis identifies distinct cardiac cell populations and reveals enrichment of DNA damage response and extracellular matrix pathways. (A)** Uniform Manifold Approximation and Projection (UMAP) plots displaying the integrated single-cell landscape ('Combined') and the distribution of cells originating from different sample conditions/locations ('HF_LA_CM', 'HF_LA_NCM', 'HF_LV_CM', 'HF_LV_NCM'). **(B)** Heatmap illustrating the expression scores of marker genes used to define initial cell clusters (Clusters 0-7) and assign preliminary cell type identities (Labels) such as Endothelial cells, Cardiomyocytes, and Tissue stem cells. **(C)** Heatmap showing the Z-score normalized expression of the top differentially expressed genes across all identified final cell clusters (Identity 0-13), highlighting gene signatures that distinguish each cluster. **(D)** UMAP plot displaying the identified cell clusters (Clusters 0-7) mapped onto the two-dimensional embedding space. **(E)** UMAP plot showing the distribution of major annotated cell types, including Tissue stem cells, Endothelial cells, Smooth muscle cells, and Macrophages. **(F)** Heatmap depicting the Z-score normalized expression of canonical marker genes across the major annotated cell types, confirming cell identity assignments. **(G)** Bubble plot summarizing results from pathway enrichment analysis. The plot displays enriched pathways related to DNA Damage Response (DDR), DNA replication, TGF-beta signaling, and extracellular matrix regulation. The x-axis represents the Rich Factor, bubble size corresponds to the number of genes (GeneNumber) in the pathway, and color intensity indicates statistical significance.

Following the initial processing and clustering of the single-cell RNA sequencing (scRNA-seq) data (Figure 6), we proceeded to identify and characterize the major cell populations present. Visualization using Uniform Manifold Approximation and Projection (UMAP) revealed distinct cell clusters, with contributions from different experimental conditions or sample origins clearly delineated (Figure 7A-D). Based on the expression of established marker genes, we annotated the primary cell clusters (Figure 7B), identifying populations including endothelial cells, cardiomyocytes, tissue stem cells, macrophages, and smooth muscle cells, whose spatial distribution is shown on the UMAP plot (Figure 7E). Heatmaps confirmed the expression of key marker genes defining these annotated cell types (Figure 7F) and highlighted the top differentially expressed genes characterizing each distinct cluster (Figure 7C). Importantly, functional enrichment analysis performed revealed significant enrichment of pathways central to our investigation (Figure 7G). Pathways related to DNA Damage Response (DDR), such as "DNA damage response" and "ATM-dependent DNA damage response," along with "DNA replication," were significantly enriched. Concurrently, pathways associated with fibrosis and extracellular matrix (ECM) remodeling, including the "TGF-beta signaling pathway" and "TGF-beta regulation of extracellular matrix," were also prominently enriched. These single-cell level findings, demonstrating the co-enrichment of DDR and ECM/fibrosis-related pathways within the cardiac cellular landscape, provide a context for understanding the potential role of genes like *IFI16* (previously observed to be upregulated in bulk DCM samples and associated with ECM organization) within specific cell populations contributing to DCM pathology.

### Elevated IFI16 expression associates with extracellular matrix remodeling pathways in DCM

**Figure 8:**
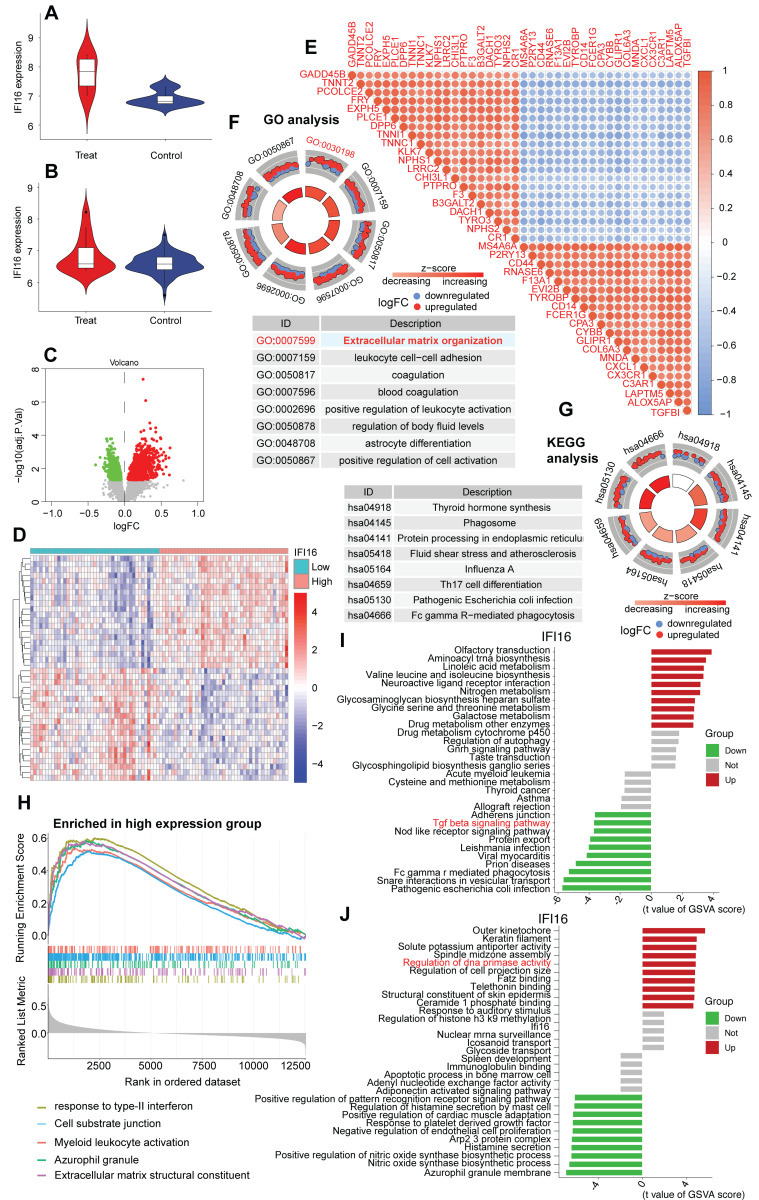
** Elevated IFI16 expression is associated with extracellular matrix remodeling pathways in human cardiac scRNA-seq data (GSE121893). (A, B)** Violin plots demonstrating significantly higher expression levels of *IFI16* in diseased ('Treat'/DCM) compared to Control samples derived from the GSE121893 dataset. **(C)** Volcano plot visualizing differentially expressed genes (DEGs) between sample groups stratified by high versus low *IFI16* expression. Red points indicate significantly upregulated genes; green points indicate significantly downregulated genes in the *IFI16*-High group. **(D)** Heatmap displaying the Z-score normalized expression levels of significant DEGs across samples grouped by low versus high *IFI16* expression, illustrating distinct transcriptomic profiles. **(E)** Correlation heatmap showing pairwise Pearson correlation coefficients between the expression levels of *IFI16* and selected key genes, including those involved in extracellular matrix (ECM) remodeling (*e.g., COL1A1, COL3A1, FN1, TGFB1*). Red indicates positive correlation; blue indicates negative correlation. **(F)** Circular plot summarizing Gene Ontology (GO) enrichment analysis results for DEGs identified between *IFI16*-High and *IFI16*-Low groups. Enriched Biological Process terms are shown, with "Extracellular matrix organization" highlighted. Segments may indicate logFC direction of constituent genes. **(G)** Circular plot summarizing Kyoto Encyclopedia of Genes and Genomes (KEGG) pathway enrichment analysis results for the DEGs. Enriched pathways are displayed. **(H)** Gene Set Enrichment Analysis (GSEA) plot showing positive enrichment of an ECM-related gene set in the *IFI16*-High expression group compared to the *IFI16*-Low group. **(I, J)** Bar plots displaying Gene Set Variation Analysis (GSVA) results, illustrating the association between *IFI16* expression levels and the activity scores of various gene sets categorized by Biological Process (I) and Molecular Function (J). Red bars indicate positive association; green bars indicate negative association.

To further explore the role of IFI16 within a high-resolution cellular context, we utilized the publicly available single-cell RNA sequencing (scRNA-seq) dataset GSE121893, derived from human left atrial and ventricular heart tissues, including samples from patients with dilated cardiomyopathy (DCM). Consistent with findings in bulk datasets, analysis within GSE121893 confirmed the significant upregulation of the DNA sensor *IFI16* in diseased samples/conditions compared to controls (Figure 8A, B). To investigate the functional consequences associated with this upregulation within the GSE121893 dataset, we stratified cells based on *IFI16* expression levels and performed differential gene expression analysis. This identified numerous significantly differentially expressed genes (DEGs) between *IFI16*-High and *IFI16*-Low groups, as visualized by volcano plot (Figure 8C) and heatmap (Figure 8D). Notably, correlation analysis within this scRNA-seq data revealed strong positive co-expression between *IFI16* and key genes involved in extracellular matrix (ECM) remodeling and fibrosis, such as *COL1A1*, *COL3A1*, *FN1*, and *TGFB1* (Figure 8E). Functional enrichment analysis of these DEGs using Gene Ontology (GO) showed highly significant enrichment for 'Extracellular matrix organization' (GO:0030198), among other terms (Figure 8F), while KEGG analysis highlighted pathways including phagosome activity (Figure 8G). Furthermore, Gene Set Enrichment Analysis (GSEA) comparing the *IFI16*-High versus *IFI16*-Low profiles derived from the GSE121893 data demonstrated significant positive enrichment for gene sets related to ECM components in the *IFI16*-High group (Figure 8H). Gene Set Variation Analysis (GSVA) further explored these connections, revealing significant associations between *IFI16* expression levels and the activity scores of numerous biological processes and molecular functions within the GSE121893 dataset (Figure 8I, J). Taken together, analysis of the GSE121893 scRNA-seq data reinforces that elevated *IFI16* expression is associated with a transcriptomic signature strongly linked to ECM organization and fibrosis pathways at the cellular level in human heart tissue, suggesting a role for IFI16 in connecting DNA damage sensing to adverse cardiac remodeling in DCM.

### Inhibition of DDR with NU7441 reduces IFI16 levels and ameliorates cardiac dysfunction in doxorubicin-induced DCM

**Figure 9:**
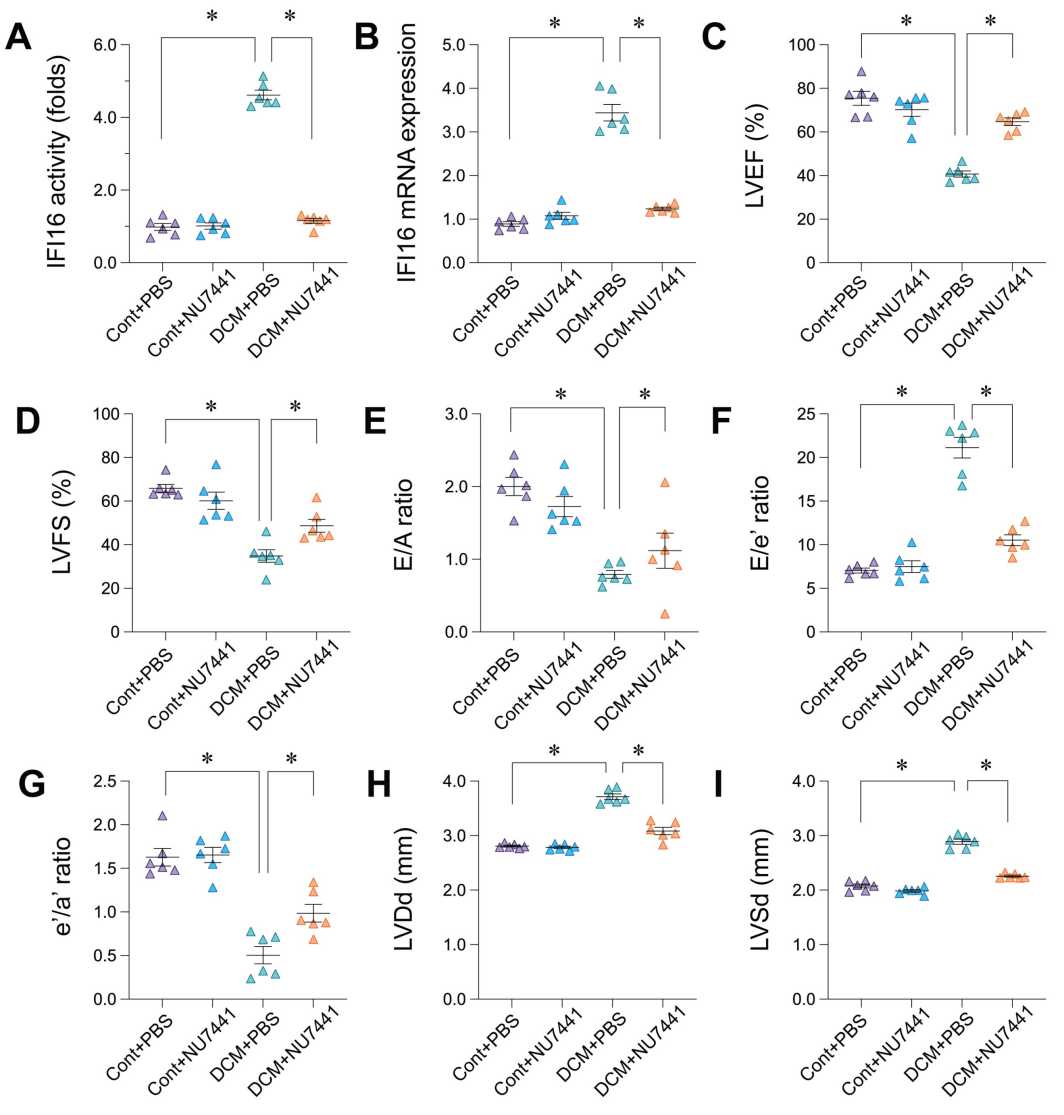
** Pharmacological inhibition of DNA-PKcs with NU7441 attenuates IFI16 upregulation and ameliorates cardiac dysfunction in doxorubicin-induced dilated cardiomyopathy (DCM). (A, B)** Relative IFI16 activity levels (A) and mRNA expression levels (B) in cardiac tissue from the four experimental groups: Control mice treated with PBS (Cont+PBS), Control mice treated with NU7441 (Cont+NU7441), DCM mice treated with PBS (DCM+PBS), and DCM mice treated with NU7441 (DCM+NU7441). Data are presented as fold change relative to the Cont+PBS group. **(C-I)** Echocardiographic assessment of cardiac function and dimensions in the four experimental groups. Parameters measured include: (C) Left Ventricular Ejection Fraction (LVEF, %), (D) Left Ventricular Fractional Shortening (LVFS, %), (E) Ratio of early (E) to late (A) diastolic filling velocity (E/A ratio), (F) Ratio of early diastolic filling velocity (E) to early diastolic mitral annular velocity (e') (E/e' ratio), (G) Ratio of early (e') to late (a') diastolic mitral annular velocity (e'/a' ratio), (H) Left Ventricular internal Diameter in diastole (LVDd, mm), and (I) Left Ventricular internal Diameter in systole (LVSd, mm). For all panels, individual data points represent biological or technical replicates as appropriate. Data are shown as mean ±SEM. *Indicates significant difference (p < 0.05) between the groups connected by the bracket/line.

To investigate whether inhibiting the DNA Damage Response (DDR) could ameliorate cardiac dysfunction and affect *IFI16* levels in our doxorubicin-induced Dilated Cardiomyopathy (DCM) model, mice were treated with the DNA-PKcs inhibitor NU7441 or vehicle (PBS). As anticipated, DCM mice treated with PBS (DCM+PBS) exhibited significantly elevated *IFI16* activity and mRNA expression compared to control mice treated with PBS (Cont+PBS) (Figure 9A, B). Notably, concurrent treatment with NU7441 (DCM+NU7441) significantly attenuated the doxorubicin-induced increase in both *IFI16* activity and mRNA levels compared to the DCM+PBS group (Figure 9A, B). NU7441 treatment did not alter basal *IFI16* levels in control animals (Cont+NU7441 vs Cont+PBS). Functionally, echocardiography confirmed severe cardiac impairment in the DCM+PBS group, characterized by significantly reduced left ventricular ejection fraction (LVEF, Figure 9C) and fractional shortening (LVFS, Figure 9D), impaired diastolic function (indicated by significantly decreased E/A and e'/a' ratios, and increased E/e' ratio; Figure 9E, G, F respectively), and cardiac dilation (increased LVDd and LVSd; Figure 9H, I) compared to Cont+PBS animals. Remarkably, administration of NU7441 significantly mitigated the cardiac dysfunction induced by doxorubicin. Compared to the DCM+PBS group, DCM+NU7441 mice displayed significantly improved LVEF and LVFS, ameliorated diastolic function parameters, and reduced left ventricular diastolic and systolic dimensions (LVDd, LVSd) (Figure 9C-I). NU7441 administration did not significantly affect cardiac function parameters in control animals. These results indicate that pharmacological inhibition of DDR via NU7441 significantly reduces *IFI16* upregulation and ameliorates cardiac dysfunction and adverse remodeling in the doxorubicin-induced DCM model.

### Inhibition of DDR in vivo and IFI16 knockdown *in vitro* attenuate pro-fibrotic and cell damage markers

**Figure 10:**
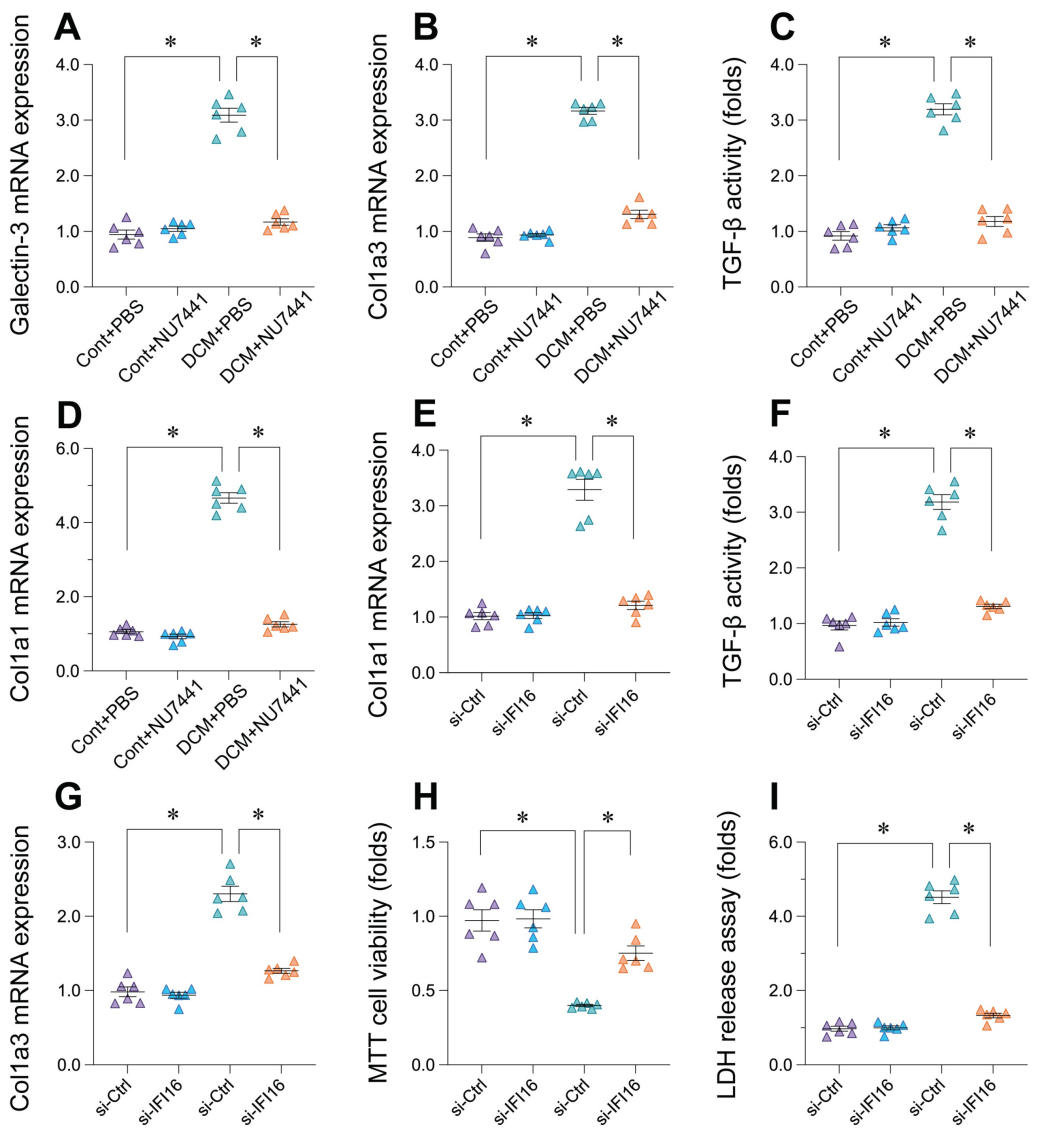
** Inhibition of DDR pathway or IFI16 knockdown reduces markers of cardiac fibrosis and cell injury. (A-D)** Effects of DNA-PKcs inhibitor NU7441 on markers of cardiac fibrosis in the doxorubicin-induced mouse model of Dilated Cardiomyopathy (DCM). Relative mRNA expression levels of (A) *Galectin-3*, (B) *Col1a3*, (D) *Col1a1*, and relative activity levels of (C) TGF-β were measured in cardiac tissue from four experimental groups: Control mice treated with PBS (Cont+PBS), Control mice treated with NU7441 (Cont+NU7441), DCM mice treated with PBS (DCM+PBS), and DCM mice treated with NU7441 (DCM+NU7441). Data are presented as fold change relative to the Cont+PBS group. **(E-I)** Effects of siRNA-mediated knockdown of *IFI16* (*si-IFI16* used in label, likely intended *si-IFI16*) compared to control siRNA (si-Ctrl) on markers of fibrosis, cell viability, and cell injury *in vitro*. Measurements include: Relative mRNA expression levels of (E) *Col1a1* and (G) *Col1a3*; (F) Relative TGF-β activity; (H) Relative cell viability assessed by MTT assay; and (I) Relative cell injury assessed by LDH release assay. Data are presented as fold change relative to the si-Ctrl group. For all panels, individual data points represent biological or technical replicates as appropriate. Data are shown as mean ±SEM. *Indicates significant difference (p < 0.05) between the groups connected by the bracket/line.

Following the observation that DDR inhibition with NU7441 improved cardiac function and reduced *IFI16* levels *in vivo*, we further investigated the effects of targeting the DDR pathway and *IFI16* on cardiac fibrosis and injury markers. In the doxorubicin-induced DCM mouse model, treatment with NU7441 significantly reduced the cardiac expression of key fibrosis-related markers compared to vehicle-treated DCM animals (DCM+PBS). Specifically, NU7441 administration resulted in significantly lower mRNA levels of *Galectin-3*, *Col1a3*, and *Col1a1*, as well as decreased TGF-β activity (Figure 10A-D). To directly assess the contribution of *IFI16*, we utilized siRNA-mediated knockdown *in vitro*. Compared to control siRNA (si-Ctrl), cells with *IFI16* knockdown (si-IFI16) exhibited significantly reduced mRNA expression of *Col1a1* and *Col1a3*, along with lower TGF-β activity (Figure 10E-G). Moreover, silencing *IFI16* led to a significant increase in cell viability as assessed by MTT assay (Figure 10H) and a significant decrease in cell injury indicated by reduced lactate dehydrogenase (LDH) release (Figure 10I). Collectively, these findings demonstrate that both pharmacological inhibition of DDR *in vivo* and direct knockdown of *IFI16 in vitro* can suppress pro-fibrotic markers. Furthermore, reducing *IFI16* levels directly mitigated markers of cell injury and enhanced viability *in vitro*, supporting a detrimental role for the DDR-IFI16 axis in contributing to both fibrosis and cellular damage in the context of cardiomyopathy.

## Discussion

Dilated cardiomyopathy (DCM) represents a major clinical challenge, characterized by progressive cardiac dysfunction and adverse remodeling, often culminating in heart failure. While diverse etiologies contribute to DCM, converging evidence suggests that compromised genomic integrity and the ensuing DNA damage response (DDR) play a significant, potentially unifying role in its pathogenesis. Our study provides compelling multi-level evidence, spanning human iPSC-derived cardiomyocytes (iPSC-CMs), *ex vivo* human heart tissue, *in vivo* murine models, and single-cell transcriptomics, that establishes a critical pathogenic axis linking DDR activation to interferon-inducible protein 16 (IFI16)-mediated extracellular matrix (ECM) remodeling in DCM. We demonstrate that IFI16, a known DNA sensor, acts as a pivotal mediator coupling nuclear stress signals to pro-fibrotic pathways, and crucially, that targeting the upstream DDR pathway can attenuate IFI16 induction and ameliorate cardiac dysfunction.

Emerging evidence points towards a significant connection between defects in the DDR and the development of DCM. The DDR is a critical cellular mechanism for maintaining genomic stability, and its impairment has been implicated in the pathogenesis of various diseases, including potentially those affecting the heart 10. Given the genetic heterogeneity of DCM, it is plausible that mutations in genes associated with DCM could directly or indirectly impact the efficiency and fidelity of DDR pathways 69. Our investigation consistently revealed robust activation of DDR pathways across disparate models of DCM. In human iPSC-CMs modeling DCM, we observed significant enrichment of cytosolic DNA sensing pathways alongside broad inflammatory signaling cascades, consistent with cellular stress responses. Intriguingly, these *in vitro* models also exhibited upregulation of DNA replication and cell cycle control pathways, potentially reflecting an aberrant proliferative or stress-induced attempt at repair in pluripotent stem cell derivatives 70-73. In contrast, analysis of *ex vivo* human heart tissue from patients with advanced DCM corroborated the activation of DNA sensing and damage pathways but revealed a significant *suppression* of DNA replication 74-77. This divergence likely reflects differences between the *in vitro* stem cell model and the complex, terminally differentiated environment of the failing adult human heart, where suppression of replication might indicate cell cycle arrest or senescence driven by persistent damage. Nonetheless, the common thread across these platforms was the clear activation of DDR sensing mechanisms. We further validated this experimentally in a doxorubicin-induced murine DCM model, demonstrating significant increases in the DNA double-strand break marker γ-H2AX, checkpoint kinase Chk2, multiple DNA repair pathway components (Ogg1, Xpc, Brca1, Ku70), and downstream effectors like p21 and Bax 78-81. These findings align with previous reports showing increased DNA damage in DCM patient biopsies and its correlation with prognosis and genotype, solidifying DDR activation as a core feature of the disease.

A particularly strong link has been observed in the context of mutations in the *LMNA* gene, a well-known cause of severe and progressive DCM 69. Studies 82-85 have demonstrated that *LMNA* mutations, which disrupt the structural integrity of the nuclear envelope, can lead to increased DNA damage and the activation of DDR pathways within cardiac myocytes 69. Research has shown that the deletion of the *Lmna* gene in fibroblasts can cause senescence-associated DCM by activating the double-stranded DNA damage response 86. This underscores the critical role of nuclear envelope integrity in maintaining genomic stability and preventing cardiac disease. Beyond genetic mutations, cellular stress and the accumulation of DNA damage appear to be significant factors in the development and progression of DCM across various etiologies. Increased DNA damage and activation of DDR pathways have been observed in cardiovascular disease and heart failure. Conditions like oxidative stress can elevate internal DNA stressors in cardiomyopathies 3. Notably, the extent of myocardial DNA damage has been found to correlate with treatment response and prognosis in DCM patients 5. This suggests that the accumulation of DNA damage in cardiomyocytes, whether due to impaired repair or increased stress, is a critical aspect of DCM pathogenesis.

IFI16 is not only a sensor of foreign DNA but also plays an active role within the DDR pathways 87. The expression of the *IFI16* gene is itself induced by the activation of the DDR in response to DNA damage, indicating its involvement in this cellular defense mechanism 87. IFI16 interacts with key DDR proteins, most notably the tumor suppressor p53 87. This interaction is crucial for amplifying the DDR, as IFI16 promotes the phosphorylation and activation of p53 in response to DNA damage 87. By enhancing p53 activity, IFI16 contributes to cell cycle arrest and apoptosis, critical processes for preventing the propagation of damaged cells 87. Interestingly, IFI16 has also been shown to inhibit DNA repair in certain contexts, potentially potentiating the effects of type-I interferons. This suggests a complex and context-dependent role for IFI16 in modulating the cellular response to DNA damage. Furthermore, IFI16 can activate ATM-p53 DDR signaling and inhibit telomerase activity 87, and it is involved in the activation of the inflammasome in response to DNA damage, linking the DDR to inflammatory pathways 87. IFI16 shows a preference for binding to quadruplex DNA structures over other DNA forms. This binding affinity suggests a role for IFI16 in stabilizing these structures, which may be relevant in DNA damage responses and cellular processes such as proliferation 88.

A key advance of our study is the identification of IFI16 as a central player connecting DDR activation to detrimental cardiac remodeling 89, 90. While IFI16 is recognized for its role in innate immunity and sensing viral/bacterial DNA, and its interaction with DDR components like p53 is established, its specific function in linking DDR to cardiac fibrosis has remained largely unexplored. Our Weighted Gene Co-expression Network Analysis (WGCNA) on human DCM tissue was instrumental, identifying a gene module highly correlated with DCM status that was co-enriched for both DDR/DNA replication *and* critical ECM/fibrosis pathways, including TGF-β signaling. This suggested an integrated network response. Subsequent analysis of human cardiac single-cell RNA-seq data provided cellular resolution, confirming significant *IFI16* upregulation in DCM conditions. Strikingly, stratifying cells by *IFI16* expression revealed that high *IFI16* levels were strongly associated with a transcriptomic signature enriched for 'Extracellular matrix organization' and correlated positively with key fibrotic genes like *COL1A1*, *COL3A1*, *FN1*, and *TGFB1*. This robust correlation at the single-cell level strongly implicates IFI16 in orchestrating pro-fibrotic gene programs within the diseased heart 91-95, moving beyond its canonical role as a purely immune sensor in this context 96-99. This contrasts with studies primarily focusing on other DNA sensors like cGAS in cardiac pathology and highlights a potentially distinct or complementary role for IFI16 in driving cardiac fibrosis secondary to DNA damage.

Research specifically examining the direct relationship between IFI16 and DCM is still an emerging area. However, several studies provide insights into potential connections 100-103. Transcriptomic analyses of heart tissue from patients with *LMNA*-related DCM have revealed increased DNA damage and activation of cytosolic pattern recognition pathways, which are thought to involve DNA sensors such as IFI16 104, 105. These findings suggest that in the context of *LMNA* mutations, the resulting nuclear instability might lead to the release of DNA into the cytoplasm, where it could be sensed by IFI16, potentially triggering downstream inflammatory signaling 106, 107. IFI16's involvement in PANoptosis, a pro-inflammatory form of programmed cell death, also suggests a potential link to DCM 9. Given the role of inflammation and cardiomyocyte death in DCM pathogenesis, IFI16's participation in these processes could be significant. One study indicated that the activation of cytosolic pattern recognition in cardiomyocytes in *LMNA*-related DCM was independent of cGAS, another major cytosolic DNA sensor, implying that other sensors, possibly including IFI16, might be more relevant in this specific context 108, 109. While direct and conclusive evidence specifically linking IFI16 to DCM is still developing, these studies, along with IFI16's known roles in inflammation and DNA sensing in other cardiovascular conditions like abdominal aortic aneurysm and in response to viral infections, suggest a potential involvement in the pathogenesis of DCM across different etiologies 110, 111.

The causal link within the proposed DDR-IFI16-ECM axis was substantiated through targeted interventions. Pharmacological inhibition of DNA-PKcs, a key DDR kinase, using NU7441 in our doxorubicin-induced DCM model yielded remarkable results 112-115. NU7441 treatment not only confirmed the dependence of *IFI16* upregulation on DDR signaling by significantly reducing its expression and activity but also led to a significant amelioration of cardiac dysfunction 116-119. This included improvements in systolic function, diastolic parameters, and a reduction in adverse ventricular remodeling. Furthermore, this functional recovery was associated with a significant reduction in cardiac fibrosis markers, including *Galectin-3*, *Col1a3*, *Col1a1*, and TGF-β activity, upon NU7441 treatment 120, 121. Complementing these *in vivo* findings, direct siRNA-mediated knockdown of *IFI16 in vitro* mirrored the anti-fibrotic effects, significantly reducing *Col1a1*, *Col1a3*, and TGF-β activity. Importantly, *IFI16* silencing also directly enhanced cardiomyocyte viability and reduced cell injury markers, supporting a direct detrimental role of IFI16 beyond just promoting fibrosis 122, 123. Together, these interventional data provide strong functional evidence that disrupting the DDR-IFI16 cascade, either upstream via DDR inhibition or directly at the level of IFI16, can mitigate both cardiac fibrosis and cardiomyocyte injury, leading to improved cardiac function.

This study significantly advances our understanding by proposing a novel mechanism where nuclear stress, manifested as DNA damage, is transduced via the IFI16 sensor to drive pathological ECM remodeling—a hallmark of DCM progression 124-127. The identification of this specific DDR-IFI16-ECM axis offers a potential therapeutic vulnerability. Our demonstration that inhibiting DNA-PKcs with NU7441 effectively counters both IFI16 induction and DCM progression *in vivo* highlights the potential utility of targeting DDR components as a therapeutic strategy 128, 129. While DDR inhibitors are primarily explored in oncology, our findings suggest their potential repurposing for cardiac diseases characterized by significant DNA damage, although careful consideration of systemic and off-target effects would be paramount. Alternatively, developing strategies to specifically target IFI16 activity or its downstream signaling pertinent to ECM regulation in the heart could offer a more focused therapeutic approach.

Certain limitations warrant consideration 130-133. Our *in vivo* work primarily utilized a doxorubicin-induced model, which, while relevant for chemotherapy-induced cardiomyopathy and robustly activating DDR, may not fully recapitulate the heterogeneity of human DCM etiologies (e.g., genetic, viral). Future studies validating these findings in genetic models (like *LMNA*-mutant mice) or other stress-induced DCM models would strengthen the conclusions 134-137. While our scRNA-seq analysis implicated multiple cell types in expressing DDR/ECM pathway components, further investigation using cell-type-specific manipulations is needed to dissect the precise contribution of cardiomyocytes versus fibroblasts or immune cells to the IFI16-mediated fibrotic response 138, 139. Elucidating the exact molecular steps connecting IFI16 activation to TGF-β pathway induction and collagen synthesis represents a critical next step.

In conclusion, our comprehensive study integrates bioinformatics, experimental models, and functional interventions to uncover a pathogenic cascade in DCM initiated by DNA damage response activation, mediated by the DNA sensor IFI16, and culminating in detrimental ECM remodeling and cardiac dysfunction. We identify IFI16 as a critical node linking nuclear stress to fibrosis and demonstrate that interrupting this axis via DDR inhibition holds therapeutic promise. These findings not only provide novel mechanistic insights into DCM pathogenesis but also highlight the DDR-IFI16 interface as a potential target for future therapeutic development aimed at halting or reversing adverse cardiac remodeling in patients with dilated cardiomyopathy.
